# Safety net or social barrier? Social networks and barriers to monitoring type 2 diabetes management among Black/African American men

**DOI:** 10.1016/j.pmedr.2025.103137

**Published:** 2025-06-12

**Authors:** Tyler Prochnow, Megan S. Patterson, Jeong-Hui Park, Ledric D. Sherman, Matthew Lee Smith

**Affiliations:** a School of Public Health, Texas A&M Health Science Center, 212 Adriance Lab Rd., College Station, TX 77843, USA; b Center for Health Equity and Evaluation Research, Texas A&M University, College Station, TX 77843, USA; c Center for Community Health and Aging, Texas A&M University, College Station, TX 77843, USA

**Keywords:** Type 2 diabetes, Social networks, Barriers, Black/African American men, Disease management

## Abstract

**Objective:**

Type 2 diabetes (T2D) disproportionately affects Black/African American men, experiencing higher rates of complications and unique barriers to disease management. While social support is known to influence health outcomes, limited research has examined how characteristics of social networks relate to T2D management barriers in this population. This study investigated associations between social network characteristics and barriers to T2D management among Black/African American men.

**Methods:**

Black/African American men in the United States with T2D (*n* = 1225) were recruited through an online panel in 2024. Participants completed a comprehensive survey assessing social networks, barriers to T2D management using the Diabetes Care Profile, and demographic characteristics. Multiple linear regression analyses examined associations between network characteristics (interactions, social norms, composition, support, and structure) and barriers while controlling for demographic variables.

**Results:**

Significant associations emerged between social network characteristics and T2D management barriers (R^2^ = 0.172, *p* < .001). Diabetes-specific discussions (β = 0.224, p < .001) and presence of other individuals with T2D in one's network (β = 0.065, *p* = .027) were positively associated with reported barriers, while perceived network member physical activity (β = −0.143, *p* = .002) and having very supportive network members (β = −0.268, *p* < .001) were negatively associated with barriers. Network size and heterogeneity were not significant.

**Conclusions:**

These findings highlight the complex role of social networks in T2D management among Black/African American men, emphasizing the importance of support quality over network size. Interventions should focus on enhancing existing support relationships and leveraging positive health behavior modeling within networks rather than simply expanding social connections. Future research should examine these relationships longitudinally to inform culturally appropriate interventions.

## Highlights

• Analysis of 1225 Black men revealed quality of social support matters more than network size.
• Having highly supportive network members reduced diabetes management barriers significantly.
• Physical activity modeling in social networks linked to fewer management challenges.
• Diabetes-specific discussions increased when individuals faced management difficulties.
• Study used innovative name generator approach to map diabetes-specific social networks.

## 1 Introduction

Chronic disease management in the United States continues to present significant challenges to public health, with Type 2 diabetes (T2D) emerging as a particularly complex condition that intersects with persistent health disparities. Approximately 37.3 million Americans currently live with diabetes (Centers for Disease Control and Prevention (CDC), 2022). Black/African American communities experience a 60 % higher diagnosis rate compared to their non-Hispanic White counterparts (Beckles, 2016). This disparity becomes even more pronounced among Black/African American men, who face higher rates of T2D and experience more severe complications including cardiovascular disease, kidney failure, and lower-limb amputations (Assari et al., 2020). The intersection of these health outcomes with social determinants of health, cultural factors, and systemic barriers creates a unique challenge that requires careful examination of both individual and social factors affecting disease management (Bhattacharya, 2024; High, 2022).

T2D management encompasses daily decisions, behavioral modifications, and consistent monitoring protocols that must integrate with existing social and cultural frameworks (Powers et al., 2020; Smith et al., 2022). These routines include regular blood glucose monitoring, medication adherence, dietary modifications, physical activity, and ongoing healthcare engagement - each impacted by social relationships and culture, and each representing a potential point where management barriers may arise (Alexandre et al., 2021; Nam et al., 2011). Research indicates Black/African American men face unique challenges in healthcare engagement, influenced by historical experiences with healthcare systems, cultural perspectives on health, and masculine identity norms (Gilbert et al., 2016; Powell et al., 2016). These challenges particularly affect blood glucose monitoring adherence, where practical and psychosocial barriers intersect (Abbott et al., 2021; Sherman and McKyer, 2015).

From a theoretical perspective, the social ecological model would posit interpersonal relationships and community structures influence health management behaviors (McLeroy et al., 1988). This theoretical perspective suggests barriers to effective health management may emerge from social obligations, cultural expectations, and community norms that may conflict with recommended medical protocols (Gholamnejad et al., 2018). Further, the network episode model examines how individuals experiencing health challenges may engage differently with their social networks to help manage these challenges (Perry and Pescosolido, 2015). Complementing this approach, social capital theory illuminates how social networks can provide essential resources, information, and support that facilitate disease management (Lin, 2017). Together, these frameworks highlight the nuanced ways social connections simultaneously support and complicate T2D management efforts.

The role of social networks in health management has emerged as a critical area of investigation, particularly as researchers recognize the limitations of individually-focused interventions (Sherman and Williams, 2018; Valente, 2017). Social networks function as complex systems that can either facilitate or impede health behaviors through multiple mechanisms including information dissemination, resource sharing, emotional support, and social norm establishment (Gatlin et al., 2017; Perry and Pescosolido, 2015; Prochnow et al., 2025a; Valente, 2017). For Black/African American men, these networks often operate within distinct cultural contexts emphasizing community relationships, collective well-being, and social obligations (Griffith et al., 2016; Griffith et al., 2012; Taylor et al., 2013). Specific social network characteristics influence health outcomes, including interaction patterns, social norms, network composition, and support quality (Hunter et al., 2019; Prochnow and Patterson, 2022). These characteristics take on particular significance for Black/African American men with T2D, as they navigate disease management within social contexts shaped by cultural traditions, community ties, and experiences with healthcare systems (Cheatham et al., 2008; Hammond et al., 2010). Understanding how these network characteristics relate to specific management barriers could provide crucial insights for intervention development.

Despite growing recognition of social networks' importance in health management, their relationship to T2D management barriers among Black/African American men remains understudied. While research has documented various individual-level barriers and the general importance of social support, limited attention has been paid to how specific network characteristics might influence monitoring barriers in this population (Hawkins, 2019; Sherman and Williams, 2018). The present study addresses this critical research gap by examining associations between social network characteristics and barriers to T2D monitoring among Black/African American men. These insights could contribute to reducing persistent disparities in diabetes outcomes and improving the effectiveness of T2D management support for this underserved population.

## 2 Methods

### 2.1 Study design

This cross-sectional study used a Qualtrics survey (February–June 2024) to assess social networks and T2D-related behaviors among Black/African American men residing in the United States. The sample was obtained through Cloud Research, which enabled concentrated recruitment of this specific population. A complete description of the study design can be found elsewhere (Prochnow et al., 2025b).

### 2.2 Participants and procedures

The study sample consisted of 1225 Black/African American men with T2D. Inclusion criteria were: (1) self-identification as Black/African American; (2) identify as male; (3) age 21 years or older; (4) self-reported T2D medical diagnosis; and (5) reside in the United States. Potential participants were directed to an internet-based Qualtrics survey link and provided with an Institutional Review Board-approved information sheet. Participation was voluntary, and respondents could withdraw at any time. Three quality checks ensured data integrity; all participants passed these checks (Curran, 2016). A total of 4184 individuals viewed the consent sheet and screening questions; however, 1604 individuals were deemed not qualified based on inclusion criteria, 706 failed a quality check, and 649 were removed based on missing data. The final study sample consisted of 1225 Black/African American men with T2D. This study was approved by XXXX Institutional Review Board (IRB2023-1311M).

### 2.3 Measures

#### 2.3.1 Social networks

A multiple name generator approach was used to elicit members of participants' social networks, following an adapted Arizona Social Support Interview Schedule (Barrera, 1980; Marin and Hampton, 2007). This comprehensive method allows for a detailed assessment of participants' personal support networks (egocentric networks) related to their T2D management (Perry and Pescosolido, 2015). Participants were prompted with questions corresponding to different forms of social interaction (e.g., people who give them advice, people they confide in, people who provide practical support, and people who make managing their T2D difficult) and asked to list individuals who fit each area. For each network member identified in the multiple name generators, participants provided comprehensive details about demographics, behaviors, relationship qualities, and interpersonal connections. For each individual network member, participants were asked to indicate their relationship type (spouse, child, parent, friend, sibling, extended family member, healthcare provider, coworker, roommate, neighbor, or other) and whether they had T2D themselves (yes, no, I don't know). Health behaviors were assessed through two key measures: perceived physical activity frequency and healthy eating habits, both were rated on a four-point scale (never, rarely, sometimes, often). Perceived supportiveness specific to diabetes management was evaluated using a four-point scale (not at all supportive, a little supportive, sometimes supportive, very supportive). Contact frequency with each individual was measured using a six-point scale ranging from several times daily to never.

Several network-level variables were calculated to characterize the social environment, examining factors such as network size, the proportion of network members by relationship type (i.e., spouse, child, parent, friend, other family member, health care provider), percentage of network members with T2D, relationship heterogeneity (measure of how many different relationships showed up in their network), mean communication frequency, average level of network support, the frequency of diabetes-specific discussions, and perception of members' health behaviors (eating healthy and being physically active). Due to the compositional nature of network relationship type data (percentages summing to 100 %), centered log-ratio transformations were performed on network composition variables prior to analysis (Espinoza et al., 2020). This transformation, calculated as the natural logarithm of each component divided by the geometric mean of all components, addresses the constraints and dependencies inherent in compositional data while preserving the relative relationship information.

#### 2.3.2 Barriers to monitoring practices for T2D

The Diabetes Care Profile – Monitoring Barriers and Understanding Management Practice Scales (DCP-MBUMPS) were utilized to evaluate barriers to T2D monitoring and the frequency of management practices among participants (Fitzgerald et al., 1996). Eleven items assessed the frequency of failed blood sugar tests due to various reasons, including forgetting, doubting the utility of testing, inappropriate timing or location, disliking the task, running out of test materials, cost, inconvenience, difficulty reading test results, inability to perform the test independently, infrequent changes in levels, and discomfort from finger pricks. Responses were recorded on a 5-point Likert scale: 1 = rarely, 3 = sometimes, and 5 = often. The possible scores ranged from 11 to 55 and higher scores on the scale indicate a greater presence of barriers in diabetes management.

#### 2.3.3 Covariates

Age, rurality (rural, suburban, urban, or other), educational attainment (less than high school, some college/2-year degree, 4-year degree or higher), employment status (student, employed, unemployed, retired, or unable to work), annual household income (in $25,000 USD increments), marital status (married/partnered, never married, divorced/separated, or widowed), and Body Mass Index (BMI) were adjusted in subsequent analyses.

### 2.4 Data analysis

Descriptive statistics, including frequencies, means, and standard deviations, were computed to summarize the characteristics of the participants. Multiple linear regression examined associations between network characteristics and T2D barriers, controlling for demographics (SPSS v.29; *p* < .05). Specifically, regression models included five categories of network predictors (interactions, social norms, composition, support characteristics, and structure) to examine their independent associations with barriers to diabetes management while adjusting for age, education, residential area, employment status, income, marital status, and body mass index.

## 3 Results

### 3.1 Sample characteristics

Participants averaged 41.9 years (SD = 14.5) with mean BMI of 31.0 (SD = 9.2) and resided in urban (52.4 %), suburban (36.1 %), or rural areas (11.1 %). Education varied: 34.0 % held 4-year degrees, 42.9 % some college, 23.1 % high school or less. Most were married/partnered (61.1 %) and employed (78.2 %). See Table 1 for more information.

**Table 1:** Sample characteristics of Black/African American men with type 2 diabetes in 2024 (*N* = 1225).

**Characteristic**	**Mean (SD)**	**n**	**%**
**Age (years)**	41.9 (±14.5)		
**Body Mass Index (kg/m**^**2**^**)**	31.0 (±9.2)		
**Number of Chronic Conditions**	2.5 (±1.9)		
**Residential Area**			
Urban		642	52.4
Suburban		442	36.1
Rural		136	11.1
Other		4	0.3
**Educational Attainment**			
Some high school, no diploma		20	1.6
High school diploma/GED		263	21.5
Some college, no degree		315	25.8
Technical/vocational training		43	3.5
Associates degree		166	13.6
Bachelor's degree		311	25.4
Master's degree		91	7.4
Doctoral degree		14	1.1
**Annual Household Income**			
Less than $24,999		140	11.4
$25,000–$49,999		323	26.4
$50,000–$74,999		303	24.7
$75,000–$99,999		223	18.2
$100,000–$124,999		109	8.9
$125,000–$149,999		52	4.2
More than $150,000		74	6.0
**Marital Status**			
Married/Partnered		749	61.1
Never Married		338	27.6
Divorced/Separated		108	8.8
Widowed		31	2.5
**Employment Status**			
Employed		958	78.2
Retired		119	9.7
Not Employed		72	5.9
Disabled		54	4.4
Student		23	1.9

Note: SD = Standard Deviation, GED = General Educational Development program, kg = kilograms, m = meters. Total percentages may not equal 100 due to rounding.

### 3.2 Social network characteristics and barriers to diabetes management

Networks included approximately six individuals (mean = 5.8, SD = 4.3) and contained the highest proportion of friends (18.8 %), followed by healthcare providers (17.7 %), parents (15.1 %), siblings (12.0 %), and spouses (11.7 %). Extended family members (10.6 %) and children (4.9 %) encompassed smaller proportions of participants' networks. Network composition indicated moderate relationship heterogeneity (mean = 0.8, SD = 0.4). On average, 18.6 % of network members also had T2D. A majority of network members were perceived as very supportive (64.8 %), with participants reporting high levels of overall social support (mean = 3.6, SD = 0.6).

Regression analysis yielded significant findings (R^2^ = 0.172, *p* < .001; Table 2). T2D-specific discussions (β = 0.224, p < .001) and having individuals with T2D in one's network (β = 0.065, *p* = .027) were positively associated with barriers. Greater perceived network member physical activity (β = −0.143, *p* = .002) and having very supportive network members (β = −0.268, p < .001) showed a negative association with barriers. Age was also significantly associated with barriers (β = −0.176, p < .001), with younger participants reporting more management challenges.

**Table 2:** Social network characteristics predicting barriers to diabetes management among Black/African American men with type 2 diabetes in 2024 (N = 1225).

**Network Characteristic**	**β**	***p*-value**
Network Interactions		
T2D-specific discussions	0.224	<0.001
General talk frequency	−0.090	0.117
Infrequent contact	−0.038	0.386
Social Norm		
Physical activity perception	−0.143	0.002
Healthy eating perception	0.015	0.753
Network Composition		
Percent network with T2D	0.065	0.027
Percent Healthcare providers	0.073	0.126
Percent Spouse	0.076	0.063
Percent Friend	0.065	0.136
Percent Parent	0.045	0.298
Percent Sibling	0.056	0.118
Support Characteristics		
Very supportive members	−0.268	<0.001
Mean support level	0.049	0.442
Network Structure		
Network size	−0.048	0.079
Network heterogeneity	0.029	0.298

Note: β = standardized regression coefficient, T2D = type 2 diabetes. Controlling for age, education, residential area, employment status, income, marital status, and body mass index. Model R^2^ = 0.172, *p* < .001.

## 4 Discussion

This study revealed important patterns in how social network characteristics relate to T2D management barriers among Black/African American men, with implications for theory and practice.

### 4.1 Network interactions

The strong positive association between diabetes-specific discussions and reported barriers presents an interesting paradox. Rather than indicating such discussions create barriers, this relationship likely reflects individuals experiencing more management challenges engage in more frequent diabetes-related conversations seeking support and guidance (Perry and Pescosolido, 2015; Small, 2017; Vassilev et al., 2014). This interpretation aligns with the network episode model, which suggests that individuals activate different aspects of their social networks during health challenges (Perry and Pescosolido, 2015; Small, 2017). When facing difficulties with blood glucose monitoring or other management aspects, individuals may initiate more diabetes-focused conversations with network members as a coping mechanism and problem-solving strategy. Previous research has consistently demonstrated that health-related discussions tend to increase during periods of management difficulty, particularly among individuals managing chronic conditions (Jones et al., 2008; Perry and Pescosolido, 2015; Schram et al., 2021). For Black/African American men specifically, these increased discussions may serve multiple purposes: seeking practical advice, emotional support, and validation of their experiences (Taylor et al., 2013; Vassilev et al., 2014), while navigating cultural expectations that often discourage open discussion of health vulnerabilities. Traditional masculine norms may initially create resistance to such discussions, but the severity of T2D management challenges can override these cultural constraints, leading to more frequent health-focused conversations as a necessary coping mechanism. The heightened frequency of diabetes-specific discussions may also indicate greater engagement with one's condition management, even if barriers persist. Moreover, this relationship highlights the complex role of social networks in chronic disease management. While increased discussions may signal ongoing challenges, they also represent opportunities for intervention and support. Healthcare providers and intervention designers should recognize that frequent diabetes-related discussions within social networks may serve as an indicator of individuals requiring additional support or resources, rather than viewing such discussions as a sign of optimal functioning (Jones et al., 2008; Taylor et al., 2013; Vassilev et al., 2014). This understanding could inform more nuanced approaches to leveraging social networks in diabetes management support.

### 4.2 Social norms

The significant negative relationship between perceived network member physical activity and management barriers illuminates the powerful role of social modeling in health behaviors, particularly within the context of T2D management among Black/African American men (Prochnow and Patterson, 2022). This finding builds upon seminal research on social contagion in health behaviors by demonstrating how the visible health practices of network members may influence T2D management (Christakis and Fowler, 2007). When individuals perceive their network members as physically active, they appear to encounter fewer barriers to their own T2D management, suggesting that active lifestyle modeling within social networks may help normalize and facilitate health-promoting behaviors (Prochnow and Patterson, 2022; Prochnow et al., 2020). The social cognitive mechanisms underlying this relationship suggest that network members who engage in regular physical activity provide both behavioral modeling and practical support for active lifestyles, including serving as exercise partners, sharing information about physical activity opportunities, or creating social environments where active living is valued and encouraged (Bandura, 2002; Sallis et al., 2006).

The absence of association between perceived healthy eating and barriers reveals important insights about social influence processes. The cultural significance of food and eating practices within Black/African American communities may create complex dynamics that influence how dietary behaviors are perceived and adopted (Lee et al., 2019). Cultural traditions often center around communal eating and specific food preparations that may conflict with diabetes management recommendations, while masculine identity norms may discourage men from appearing overly concerned with dietary restrictions. In contrast, physical activity may be more readily modeled and adopted because it aligns with traditional masculine values of strength and physical capability.

Further, previous research demonstrates that people often adopt and model the eating behaviors of their close social networks, specifically family connections (Leahey et al., 2015). It may be more difficult to deviate from established behavioral norms when the activity (e.g., eating) is done in group settings specifically within family/social networks compared to engaging in physical activity (Higgs, 2015; Vartanian et al., 2015). In other words, it may be challenging for people to have control over what they eat because it is so engrained in family, cultural, and social norms. While both behaviors are crucial for T2D management, the mechanisms through which social networks influence these behaviors may differ substantially, suggesting that interventions leveraging social networks might be particularly effective when focusing on physical activity as an initial target for behavior change, as it appears to have more direct social modeling effects.

### 4.3 Network composition

The positive association between the percentage of network members with T2D and reported barriers reveals complex dynamics in shared health experiences that warrant careful consideration. While previous research has emphasized the benefits of peer support in chronic disease management (Gatlin et al., 2017; Zupa et al., 2022), our findings suggest a more nuanced relationship that may actually compound management challenges. Network members with T2D can provide valuable emotional understanding and practical knowledge derived from their own experiences (Hurt et al., 2015). However, these shared health experiences may simultaneously create additional stress when network members struggle with their own management, potentially leading to collective anxiety and burden around diabetes care (Sherman and Williams, 2018). This normalization of management challenges could create a self-reinforcing cycle where barriers are viewed as inevitable rather than surmountable, potentially reducing motivation to overcome them. The shared experience of T2D within networks might also amplify the emotional toll of management, as individuals not only cope with their own health challenges but also witness and absorb the struggles of their network members.

### 4.4 Support characteristics

The strong negative association between having very supportive network members and reported barriers emerges as one of the study's most compelling findings, highlighting the crucial distinction between support quantity and quality in diabetes management. This relationship reinforces extensive research demonstrating that high-quality social support serves as a critical factor in successful diabetes self-management, particularly among Black/African American men (Hawkins, 2019; Vassilev et al., 2014; Zupa et al., 2022). The absence of a significant association with mean support level, coupled with the strong influence of having highly supportive members, suggests that the intensity and quality of support may be more valuable than the mere presence of supportive relationships in overcoming management barriers (Hawkins, 2019; Schram et al., 2021; Zupa et al., 2022). In other words, strong support at the dyadic/relational level was more important for disease management than an aggregate score of support across all network members. These highly supportive network members likely provide multiple forms of assistance, including emotional encouragement, practical help with monitoring routines, and meaningful accountability for management behaviors (Altevers et al., 2016; Schram et al., 2021). The differential impact between having very supportive members versus generally supportive networks also suggests that intervention strategies should focus on strengthening existing supportive relationships rather than simply expanding social networks (Altevers et al., 2016; Schram et al., 2021). These findings also infer the need to better understand the specific qualities of these very supportive individuals in lieu of broad social support measures. Within Black/African American cultural contexts, these highly supportive individuals often navigate complex cultural and gender dynamics, providing assistance in ways that preserve masculine identity while promoting health behaviors. Culturally speaking, it may be necessary to frame diabetes management as strength and responsibility to family rather than vulnerability or weakness.

### 4.5 Network structure

The non-significant associations between network size, heterogeneity, and barriers challenge assumptions about the benefits of larger, more diverse networks. This finding suggests that the quality and nature of social connections may be more important than network size or diversity for managing T2D among Black/African American men. Also, while size and diversity of networks may be important for information dissemination (Granovetter, 1973), it seems more supportive, close-knit ties are key for disease management in this sample.

### 4.6 Implications

The findings from this study have significant implications for healthcare providers, intervention designers, and public health practitioners working to support T2D management among Black/African American men. First, the complex relationship between diabetes-specific discussions and management barriers suggests that healthcare providers should view frequent diabetes-related conversations as potential indicators of needed support rather than a sign of a well-functioning supporting system. Providers might develop strategies to help patients activate their social networks more effectively during periods of management difficulty while providing additional resources during these challenging times. Moreover, involving close social network members who are the patient's primary support providers could foster better disease management for the patient, and taking a family-based approach to care may improve outcomes for T2D patients. Simultaneously, the potentially problematic aspects of shared T2D experiences within networks require careful consideration in peer support program design. While peer support remains valuable, interventions should include strategies to prevent the normalization of management difficulties and provide tools for managing collective stress. Perhaps most importantly, the significant impact of having very supportive network members suggests that interventions should focus on enhancing the quality of existing supportive relationships rather than simply expanding social networks. Healthcare providers might develop assessment tools to identify highly supportive network members and implement strategies to engage these individuals in treatment planning and support provision. Such approaches should carefully balance cultural sensitivity with the need to promote effective management practices by explicitly addressing how traditional masculine norms within Black/African American communities may create barriers to help-seeking and diabetes management discussions. Programs should reframe diabetes self-care as an expression of strength, responsibility, and family protection rather than weakness or vulnerability. Additionally, programs should leverage cultural values of community support and collective responsibility while respecting gender role expectations that may influence how men engage with their social networks around health issues. The cultural and gender dynamics observed in this study reflect broader patterns within Black/African American communities where traditional masculine identity intersects with cultural values around health, family responsibility, and social support. Understanding these intersections is crucial for developing interventions that work within existing cultural frameworks rather than against them.

### 4.7 Limitations

Several limitations should be considered when interpreting these results. The cross-sectional nature of the data precludes causal inference about the relationships observed. This design also does not allow for the evaluation of network dynamics or evolution over time. Self-reported data may be subject to recall and social desirability bias. Additionally, while our sample was relatively large and diverse in terms of socioeconomic status, it may not be fully representative of all Black/African American men with T2D. The use of online recruitment methods may have excluded individuals with limited internet access or technological literacy.

### 4.8 Conclusions

This study advances our understanding of how social networks influence T2D management barriers among Black/African American men, highlighting the complex interplay between social relationships and health management. The findings emphasize that the quality of social support may be more important than network size or composition in reducing management barriers. Future research should examine these relationships longitudinally and explore how interventions might effectively leverage social networks to improve T2D management outcomes in this population. These insights can inform more effective, culturally appropriate approaches to supporting T2D management among Black/African American men, potentially helping to reduce persistent health disparities in diabetes outcomes.

## CRediT authorship contribution statement

**Tyler Prochnow:** Writing – original draft, Funding acquisition, Formal analysis, Conceptualization. **Megan S. Patterson:** Writing – review & editing, Investigation, Formal analysis. **Jeong-Hui Park:** Writing – review & editing, Formal analysis. **Ledric D. Sherman:** Writing – review & editing, Funding acquisition, Conceptualization. **Matthew Lee Smith:** Writing – review & editing, Investigation, Formal analysis, Conceptualization.

## Informed consent statement

All participants viewed an informed consent page prior to the start of the study.

## Institutional review board statement

This study was approved by Texas A&M Institutional Review Board (IRB2023-1311M).

## Funding

The present study was supported by the National Institute of Minority Health and Health Disparities R21 grant (R21MD019048).

## Declaration of competing interest

The authors declare that they have no known competing financial interests or personal relationships that could have appeared to influence the work reported in this paper.

## Data Availability

Data will be made available on request.
